# Mithun (*Bos frontalis*): the neglected cattle species and their significance to ethnic communities in the Eastern Himalaya — A review

**DOI:** 10.5713/ab.21.0020

**Published:** 2021-04-23

**Authors:** Tashi Dorji, Jigme Wangdi, Yi Shaoliang, Nakul Chettri, Kesang Wangchuk

**Affiliations:** 1 International Centre for Integrated Mountain Development, GPO Box: 326, Katmandu, Nepal; 2 Department of Livestock, Ministry of Agriculture and Forest, GPO Box: 11001, Thimphu, Bhutan

**Keywords:** Eastern Himalaya, Ecological Roles, Ethnic Groups, Mithun, Socio-culture, Socio-economic

## Abstract

**Objective:**

This review consolidates the available information on the socio-economic and ecological significance of Mithun in the lives of ethnic communities in the Eastern Himalaya.

**Methods:**

Standard guidelines were followed for the review and data collection was carried out at three stages; literature search, literature screening, and literature review and analysis.

**Results:**

Records indicate a long association of Mithun with the ethnic groups. Mithun serves as a symbol of pride and local currency for barter trade in the ethnic society. Its utilities range from being used as a bride price to settling legal disputes. Several cultural festivals and local ceremonies are celebrated around this bovine. Due to its semi-wild nature, this animal also has an ecological role to conserve broad leaf sub-tropical forests. However, it remains neglected and has not received policy attention, leading to a stagnated growth. The institutions for Mithun research and development are also weak. Furthermore, the species is under threat from new diseases and habitat alteration triggered by climate change.

**Conclusion:**

Founded on the current state of knowledge, there is a need for institutional development, strengthening institutional linkages, and promoting regional cooperation among Mithun rearing countries for further research and development of this unique cattle.

## INTRODUCTION

Mithun, *Bos frontalis* [1], is an uncommon and unique bovine species, endemic to parts of the North-Eastern Himalaya. It holds an important place in the social, cultural, and religious fabrics of ethnic communities in India [2–4]. The animal is increasingly reared for meat as it fetches premium prices in the markets. The species is also critical for sustaining mountain agriculture, which is the basis for the food and nutrition security of the ethnic groups [5]. Some authors [6,7] relate the role of this indigenous animal to a nature-based solution to address issues of climate change. In recent years, Mithun has drawn the attention of historians, anthropologists, and evolutionary biologists due to its deep association with the culture of ethnic communities in the Eastern Himalaya [8].

Grouped under vulnerable species of mammals in India, the International Union for Conservation of Nature (IUCN) [9] considers Mithun conservation essential for its potential economic function in diverse climatic and difficult environmental conditions. Its unique adaptation to subtropical broadleaf, leech, and the fly-infested environment is a desirable genetic trait worthy of conservation [10]. However, this animal species has not received adequate policy and institutional support, probably due to its small population and localized distribution [11]. Consequently, there is exploitation and destruction of its habitat, posing a major threat to its already dwindling population [12].

In the face of climate change in the Eastern Himalaya, Mithun conservation could be an innovative alternative for the adaptation and resilience-building of rural people [11]. Nevertheless, initiatives to conserve a genetic resource seek a rationale beyond genetics. Policies to promote conservation require sufficient knowledge of the existing practices, without which there could be highly diverse consequences on management and the future evolution of Mithun. It requires a profound comprehension of social, cultural, economic, and environmental dimensions. Currently, documented information on Mithun is sparse and fragmented, not compiled to provide a sound basis for policy and investment priorities [13,14].

In this paper, we review the available information with the primary objective to highlight the socio-cultural, socio-economic, and ecological significance of Mithun in the ethnic society. We highlight drivers of change in Mithun husbandry and existing institutional arrangements related to its management in the region. Gaps are identified in Mithun’s research and development. To save this vital genetic resource from extinction, the study recommends vital interventions by multiple stakeholders. Transboundary cooperation is emphasized for the effective management of this unique bovine genetic resource in the Eastern Himalaya. The study findings envision drawing the attention of policymakers and provide a conservation and development base, both at the national and regional levels.

## CONDUCT OF LITERATURE REVIEW

**Figure 1:**
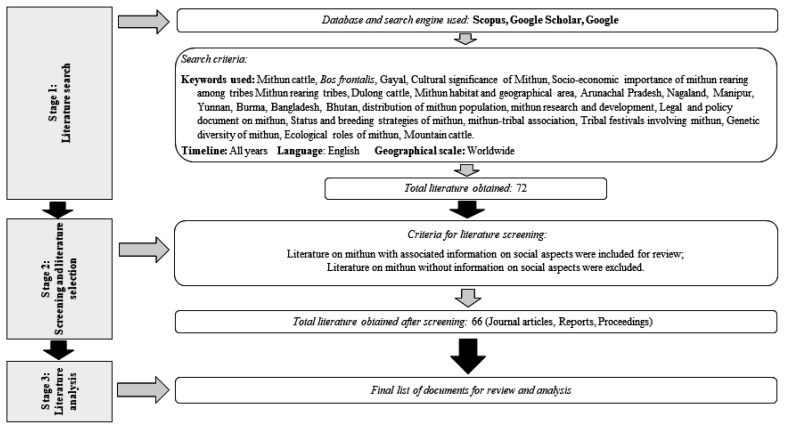
Flow diagram of methodological framework of the review.

The review of scientific literature followed the guidelines of Pullin and Steward [15]. Data collection was carried out in three stages (Figure 1).

### Stage 1: Literature search

A literature search was conducted with the bibliographic database Scopus and Google Scholar. The search in Scopus found 11 journal papers, of which 10 papers were on the genetics of Mithun; while the search in Google Scholar found 20 examples of literature with information on Mithun and associated social importance. Published literature included journal articles, reports, book chapters, and issue briefs. An additional 41 items of grey literature, including reports, websites, and books were retrieved from Google search. The entire search was guided by a set of criteria summarized below, which provided a total of 72 relevant works of literature.

- Keywords: Mithun cattle, *Bos frontalis*, gayal, cultural significance of Mithun, socio-economic importance of Mithun, Mithun rearing ethnic groups, Dulong cattle, Mithun habitat, Mithun geographical area, Arunachal Pradesh, Nagaland, Manipur, Yunnan, Burma, Bangladesh, Bhutan, distribution of Mithun population, Mithun research and development, legal and policy document on Mithun, status and breeding strategies of Mithun, Mithun-ethnic association, ethnic festivals involving Mithun, genetic diversity of Mithun, ecological roles of Mithun, and mountain cattle.
- Timeline: All years
- Language: English
- Geographical scale: Asian region

### Stage 2: Literature screening

Social aspects and the role of Mithun in ethnic lives were a pre-defined set of criteria used for literature screening and cleaning. It was applied by filtering the titles and abstracts of the collected literature. These selected articles contained information on the habitat of Mithun, the association between Mithun and ethnic groups, socio-cultural, socio-economic, ecological significance, and institutions. Screening reduced the number of literature from 72 to 66, which consisted of journal articles, book chapters, and proceedings.

### Stage 3: Literature review and analysis

Over 66 items of literature were reviewed and subjected to descriptive analysis to summarize key information under different pre-identified themes. The themes were: habitat and population distribution, socio-cultural and socio-economic significance in ethnic lives, ecological significance, drivers of change and impacts on Mithun farming, and gaps in Mithun research and development. The consolidated information was used for suggesting a way forward for Mithun’s development in the future.

## PHYLOGENY AND DOMESTICATION OF MITHUN

Mithun was domesticated more than 8,000 years ago [16] from a wild gaur, *Bos gaurus* [11,16] along the Assam-Burma border [17,18]. Rajkhowa et al [19] considered gaur, known as Indian Bison, as the wild ancestor of Mithun due to their similarities in phenotype, habitat, habits, and behavior. A few researchers postulated Mithun as the cross between gaur and domestic cattle with *banteng* blood, or Zebu’s in ancient times [8]. The idea that Mithun could be a hybrid of Siri cattle and gaur was dropped based on evidence of karyotypes and blood groups [18]. Founded on the chromosomal assessment, Winter et al [20] asserted gaur as the most probable wild ancestor of Mithun because both possessed 58 diploids (2n) chromosomes, in contrast to 60 diploids (2n) chromosomes in other cattle. Studies on molecular phylogeny and genetic diversity suggest Mithun as the direct domestication form of gaur [21], a result of moderate gene introgression from zebu and yellow cattle [22] and a cross between gaur and cattle [23].

**Figure 2:**
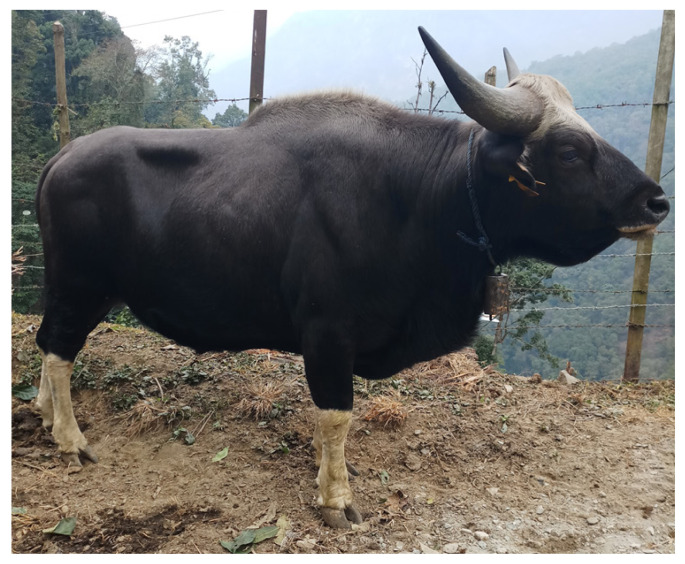
Mithun, (*Bos frontalis*), the neglected cattle species.

Mithun populations in India are categorized into four strains, namely Arunachal, Manipur, Mizoram, and Nagaland, according to the distinct physical and genetic features [24,25]. Phenotypically, Mithun has well developed and symmetrical body with distinct muscle [26], and dark reddish-brown to blackish-brown hair with white stockings (Figure 2). Adult males are larger, heavier, and have more developed body muscles and horns than females. Both sexes have horns pointed outwards and slightly curved upward with a tapering end, and the horn length ranges from 0.6 to 1.15 meters [12,27]. Faruque et al [27] reported similarities in body conformation and coat color of gayal (Mithun) from Bangledesh to those of Bhutan, China, Myanmar, Mizoram, and Nagaland of India, but different from gayal of Arunachal.

Unlike the hump of indigenous cattle, Mithun has a pro minent dorsal ridge with a strong and well-developed neck and thick muscular and folded pendulous dewlap [26]. Its legs are proportionate in size and set well apart like beef cattle, and the body length, tail length, and shoulder height range from 2.5 to 3.3 meters, 0.7 to 1.05 meters, and 0.65 to 2.2 meters, respectively for an adult animal [12]. It has a long tail that reaches up to the hock joint with a black or white switch [26].

## HABITAT AND POPULATION DISTRIBUTION OF MITHUN

**Figure 3:**
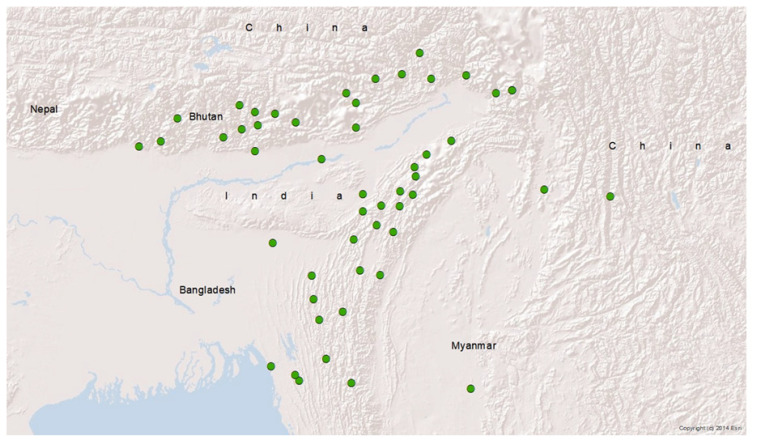
Distribution of Mithun in the southern foothills of Eastern Himalaya. Green dots represent locations of Mithun habitat.

The distribution of Mithun is localized along the southern slopes of the Eastern Himalaya (Figure 3). Unlike other livestock, Mithun is found in small numbers. Bhattacharyya et al [28] noted small numbers in the hilly tracts and areas of Myanmar, Bangladesh, China, Bhutan, and India. Geographically, the habitat of Mithun extends from low to high altitude areas and prefers a cool climate with temperatures ranging from 20°C to 30°C. Several authors concur that the Mithun’s habitat is characterized essentially by the presence of streams, ponds, and lakes, undisturbed forested areas, and hilly terrains below an altitude of 5,000 to 6,000 meters with abundant shrubs, trees, bamboos, and coarse grasses [27–30]. These observations suggest that Mithun flourishes well in thick vegetation along the riverbanks and the salt lick hotspots.

The details of the Mithun population are provided in Table 1. The actual population of Mithun is difficult to estimate due to scattered information and a lack of time-series data. Mukherjee et al [23] reported India as having the largest Mithun population (~97.57%) in the world, followed by Myanmar (0.96%, approx. 3,000 heads), China (0.96%, approx. 3,000 heads), and Bhutan (0.18%, approx. 570 heads). Other information available on Mithun population is 386,293 heads in India [31], 30,000 heads [32], and 70,000 heads [33] in Myanmar, 3,068 to 3,077 heads in China [32], 850 to 900 heads in Bangladesh [28], and 418 heads in Bhutan [34]. From this available information, the global Mithun population is estimated at 0.500 million heads.

## SOCIO-CULTURAL SIGNIFICANCE OF MITHUN

In ethnic society, the meat of Mithun is popular in marriage ceremonies, community feasts, and other important social gatherings. Considered the most delicious form of beef with a marbled texture, Project Maje [35] noted meat as an important part of wedding and Christmas celebrations. A few authors [36,37] also reported live animals being used as a gift during marriage and other social and cultural ceremonies. Moyong [30] and Chatterjee [38] observed the customary practice of offering a live animal as a bride price for marrying a girl. The importance of Mithun as a bride price is noted by Gibji [36] in the Adi tribe of Arunachal Pradesh where the marriage is considered “not finalized” until the bride’s family possesses the animal offered by the bridegroom’s family. Thus, Nemching [39] found Mithun as a basis for family, marriage, and kinship ties in the ethnic world. The ability of a man to practice polygamy in the ethnic world is primarily determined by the number of Mithun owned, which is used as a bride price [40]. On the contrary, penalties are pegged to the animal for offenses [38,39] related to social matters, particularly marriages [39,41]. It is also used to pay compensation and settle legal disputes among ethnic people [36,40]. Gambo [42] noted an intricate relation respected by ethnic communities between the animal and the myths. Thus, ethnic communities consider Mithun as a sacred animal, reared mainly for sacrifices and festivals [29,36]. Verma et al [43] provide anecdotal evidence of this animal being used in zootherapy, a traditional healing practice followed by a major ethnic group of Karbi Along district of Assam, India. In the therapy, the animal’s penis is cooked deliciously and consumed to heal the breast pain of the lactating mother [43].

In Bhutan, the Mithun breeding bulls are highly valued and considered a treasured gem. A good breeding bull is equated to ‘half the herd’, as it is a cultural belief that Mithun calves inherit good qualities from a breeding bull. For this reason, the herders are willing to pay a high price to own one for their herd. The significance of owning Mithun breeding bulls is reflected in the ways the bulls are received by the rural households in Bhutan. As a mark of welcome by a household who owns Mithun for the first time, a ceremony is performed with a traditional procession with burning incense, an offering of *Khadar* (white scarf), and *Marchang* (wine offering ceremony), followed by a serving of alcoholic drinks and tea to people witnessing the event.

Sacrifices of this bovine species are believed to bring great glory, more blessings, and appease the house god [36]. Hence, animals are sacrificed during religious festivals, and the meat of sacrificed animals fetches high prices [44]. Faruque et al [27] mentioned that the bulls are usually sold between December to February months for sacrifices during the Muslim religious festival called “Oros”, organized by the Muslim “peer”. These documented pieces of evidence highlight Mithun as a ceremonial animal having an important role in the economic, social, and cultural lives of ethnic people in Bandarban Hill and Chittagong districts in Bangladesh [3].

## DIVERSITY OF ETHNIC GROUPS ASSOCIATED WITH MITHUN

The lives of most ethnic groups in the southern foothills of Eastern Himalaya are associated with Mithun. The animal is social, friendly, and intelligent. It is generally reared by specific ethnic groups in the Mithun rearing countries of India, Bangladesh, Myanmar, China, and Bhutan. Besides having diverse roles in the lives of ethnic groups, Mithun is used as a food for festive occasions except in Bhutan. Tayo et al [45] identifies this bovine species as an important part of the social fabric and is sold at the time of adversity, indicating a strong and intricate association between the animal and ethnic communities. Among countries that rear Mithun in the region, India has the largest number of ethnic groups rearing communities Mithun. Of the total 25 Scheduled Tribes (ST), about 14 are in Arunachal Pradesh, followed by three in Manipur and one each in Mizoram and Nagaland [46]. While a small number of Mru and Marmas ethnic groups were found in Myanmar, only Zo ethnic groups from Chin, Sagaing, and Kachin were found associated with the rearing of this precious animal. Whereas in China, of the total 56 ethnic groups recorded in Yunnan province, the smallest ethnic group Dulong rears Mithun (also known as Dulong cattle), and currently, it has become an important source of cash income [47]. This review recorded about 24 ethnic groups from four countries (India, Myanmar, Bangladesh, and China) associated with Mithun farming in the region. In Bhutan, Mithun and its crossbreeds are reared by general livestock farmers, mostly owning large herds of local cattle in the migratory livestock production system. The details of ethnic groups involved in Mithun farming are presented in Table 2.

## MITHUN IN ETHNIC FESTIVALS

Festivals are the soul and essence of the socio-cultural identity of ethnic society, and in almost every festival, Mithun is sacrificed [18]. Ethnic communities across Mithun rearing countries celebrate numerous festivals and perform rituals to invoke and appease gods and goddesses for the well-being and prosperity of the animals and community. While diverse ethnic festivals are being celebrated in India, many of the festivals are celebrated by the tribes rearing Mithun, and a small number can be found in other countries. Some important Mithun-associated festivals with their salient features are presented in Table 3.

## SOCIO-ECONOMIC SIGNIFICANCE OF MITHUN IN ETHNIC COMMUNITIES

The semi-wild domesticated Mithun is reared in a free-range system. Leiden [48] reported Mithun as an efficient converter of forest biomass into fat milk (though in small quantity) and high-quality leather. In India, although the animal is not reared for milk generally, evidence suggests Mithun and its hybrids being milked in the West Kameng and Tawang districts of Arunachal Pradesh and certain parts of Mizoram [49]. A similar practice is also found in Bhutan where Jatsham (cows of Mithun crossed with cattle) are milked because of good quality milk with higher fat content, compared to other crosses and local cattle [50].

Apart from ceremonial purposes, Mithun is a source of cash income [47]. Mithun cows produce around 1 to 1.5 kg milk/d/animal [51]. Since milk has 3.4% to 17% fat, 6.8% to 22.2% solids-not-fat, and 4.4% to 9.8% protein, different milk products are prepared [52]. The hides and skin have a higher value in the tannin industry than cow leather [25]. The animal is reported to attain greater mature body weight with good beef traits than other cattle maintained under a similar environment [44]. Compared to other cattle, the meat quality is considered superior as it contains high protein in muscles and organs (14 to 19 percent), low crude fat (0.4 to 3.58 percent), and carbohydrate (0.06 to 4.97 percent) [11,24,47]. According to Yang et al [53], other characteristics that also make Mithun’s meat superior to other cattle meat are less muscle fiber diameter, greater water-holding ratio, muscle tenderness, and muscle succulence. These traits explain why this animal is reared generally for meat except in Bhutan.

Mithun is an indicator of wealth and social status in the ethnic world [17,33,36,52] and largely influences the socio-economic and cultural life of ethnic communities [36]. The number of Mithun owned is linked to the wealth of a person in ethnic society [30,53]. Thus, the owner with a large herd size is considered wealthier and prestigious [40], highlighting a positive correlation between socioeconomic status and the number of Mithun [35,53]. Chatterjee [38] reported similar practices in Myanmar where the wealth of an ethnic chief or any other individual is judged by the numbers of Mithun owned.

Mithun is generally owned by village leaders, clan heads, and wealthier people. Observations by Mahanta [40] and Project Maje [35] show that a display of an array of Mithun horns is often considered symbolic of respect, regard, and social prestige owed to the possessor by his less affluent neighbors. Considered a special animal, some ethnic communities share a strong socio-cultural and emotional relation with Mithun [45]. Acknowledging its socio-cultural importance, Arunachal Pradesh recognized this bovine species as an official animal of the state. Some authors [39,54] report live animals being used as a medium of exchange, measuring the value in the intra- and inter-ethnic relationship of Arunachal Pradesh [55–57]. It is considered a Mizo-Chin currency in Myanmar. Chatterjee [38] mentioned the existence of the barter system and the use of Mithun as a medium of exchange. The Entrepreneurs Associates in Nagaland promotes this animal as a unique micro-enterprise and eco-friendly sustainable livelihood option among farmers. As a result, Viro [58] reported enhanced income of farmers of Phek district who were able to meet education cost, build homes, purchase a vehicle, and farm machinery, which led ultimately to the conservation of forest.

## ECOLOGICAL SIGNIFICANCE OF MITHUN IN ETHNIC LIFE

As a semi-domesticated animal, Mithun is closely related to natural habitats. However, the ecological roles of Mithun are less documented. Conservationists and governments appreciate their presence in the wild, thus, protection and conservation of the habitat of Mithun are pursued to promote the protection of ecology. Leiden [48] highlighted the conservation efforts of the Government of Nagaland to protect Mithun through the initiation of forest protection to provide natural shelter to this special animal, primarily because their presence in the wild has helped to conserve some rare plant species of medicinal value. Acknowledging the unique economic contribution of Mithun, the Government of Nagaland identifies Mithun farming as a nature-based alternative to slash-and-burn agriculture, which is rampant among ethnic communities. The unregulated and widespread practices of slash and burn agriculture (that involves the felling of many trees) take a huge toll on the environment, causing acute water shortages and frequent landslides. The promotion of Mithun farming is likely to address such issues of environmental concern.

Such government initiatives have motivated farmers to designate forestland near villages as a “Mithun forest”, dedicated to the breeding of free-ranging Mithun species. As noted by Chaudhry et al [59], the Galo tribe in Arunachal Pradesh follows community-based and welfare-oriented management practices called ‘Lura’ or fencing of designated forest areas for rearing Mithun in captivity by ethnic communities. Chaudhry et al [59] also outlined forest and wildlife conservation as an important advantage of ‘Lura’. Thus, Banerjee [60] found ‘Lura’ to help reforest degraded land and restore wildlife. Reforestation promoted the growth of livestock whose grazing and manure have accelerated the growth of plants, including medicinal plants and orchids. Due to the success of the Mithun forest initiative, there has been an increasing trend to designate more lands as Mithun forests [58]. It is solely because of the existence of this semi-domesticated animal that some ethnic communities have preserved forests. If a balance is maintained between Mithun numbers and forest cover, the traditional Mithun-keeping could be the most sustainable activity with less damage on forests, as compared to herds of goats, sheep, or cattle [35].

## DRIVERS OF CHANGE AND IMPACTS ON MITHUN FARMING

Mithun husbandry has witnessed stagnated growth due to weak policy support. It is reflected in the impeded investment in infrastructure, lack of proper incentives to farmers, inadequate inputs for production, inadequate delivery of health services, and lack of effective marketing and credit facilities. The lack of strong interventions to upscale Mithun husbandry makes Mithun farming remain traditional, which is characterized by low productivity, increasing incidences of diseases, and human-wildlife conflicts. The scenario is aggravated by haphazard breeding and genetic degradation due to the unavailability of breeding bulls for replacement. The technological innovations in Mithun reproduction are limited, and development is slow in absence of dedicated institutions to coordinate and conduct Mithun research and development.

The gradual shift from subsistence to commercial-scale farming through Mithun-cattle hybridization appears to threaten the Mithun population. Forest logging and road construction activities have disturbed Mithun habitats, pushing this sacred animal into deeper forests and increasing vulnerability to attacks by predators. Generally, Mithun husbandry is impacted largely by the increasing access to alternative livelihood opportunities and growing preferences of farmers for improved cattle breeds. It is also impacted negatively by the change in governance to manage forest and grazing resources and the strict implementation of rules and regulations with an excessive focus on conservation.

Climate change is another threat to Mithun husbandry. While this indigenous cattle species has desirable traits to resist diseases and thrive under difficult conditions, it is uncertain if these traits could continue to protect it from new emerging diseases triggered by climate change. However, Mithun will likely be impacted by changes in the quality and quantity of forage available in the forest. The unexpected mortality of this bovine species could occur from extreme weather events such as landslides and floods.

## OPPORTUNITIES FOR TRANSBOUNDARY COOPERATION FOR MITHUN CONSERVATION AND DEVELOPMENT

### Ecological functions of Mithun

Studies on the ecological roles of Mithun are limited. The only evidence available is from a few authors [5,48,58,59] whose studies are confined mostly to northeast India. The current state of knowledge shows that the ecological roles of Mithun in other Mithun inhabiting areas are less explored and understood. Consequently, despite knowing Mithun’s habitat is characterized by abundant vegetation, the interaction between forests and Mithun is yet to be fully understood as one of the key processes shaping ecosystem function. Sound knowledge on the ecological roles of Mithun is fundamental for wildlife ecologists to better understand Mithun and plan sustainable habitat management. The knowledge could also reveal an unforeseen contribution of Mithun to nature-based solutions, which are relied upon for addressing emerging issues of climate change.

### Inventory and characterization of Mithun in the Eastern Himalaya

Owing to the changing lifestyle of the ethnic population and increasing sacrifices for political gains and marriage ceremonies, some authors [18,29] fear that the Mithun population might decline in the future. Lalsangpuii et al [61] note that a population decline could arise from the declining importance of Mithun amongst the younger generation of the society. Other possible causes for a gradual population decline in the future are the unavailability of certified breeding bulls locally, increasing incidences of inbreeding, a decline in grazing land, and a lack of suitable breeding and feeding management [62]. Hence, these concerns have prompted Mondal et al [63] to stress an urgent need for scientific intervention to manage properly and conserve this hill animal.

Unlike other livestock species, scientific information on the genetic characterization, breeding, and feeding management of Mithun is limited. Available investigations on genetic diversity are limited to small populations within each country. Comprehensive inventory and genetic diversity analysis of this unique bovine species across the countries could provide a sound basis for conservation and development strategies. Determining the level of inter-and-intra-population genetic diversity could guide countries to strategize on both development and conservation interventions in the changing environments.

### Mithun germplasm improvement and health management

Mithun is a polyestrous animal and breeds throughout the year and does not have definite breeding seasons. The herds move around in jungles throughout the year and breed naturally, without any human interventions [62]. Mithun cows are reported to have high reproductive efficiency with productive life ranging between 16 to 18 years and can produce one calf per year [27,63]. Nonetheless, farming is challenged with the non-availability of quality breeding bulls for a replacement that is recommended preferably once in 3 to 5 years [62]. Because of insufficient quality bulls, there could be high incidences of inbreeding and breed deterioration due to excessive use of elite bulls. Bigger-sized and good-quality animals are generally slaughtered in masses during ceremonies and festivals, leaving behind poor-quality animals for breeding purposes [61]. Thus, it is important to try out modern alternatives to address this issue. The available modern alternatives are artificial insemination technology, estrus synchronization, and embryo transfer technology. This could only be achieved through scientific rearing and exploring the production potential of Mithun using modern biotechnological tools beginning with the application of reproductive bio-techniques, feed technology, and the use of modern technologies for disease diagnosis [61].

However, to counteract the decline, Lalsangpuii et al [61] recommend focusing on breeding strategies aimed at conserving this precious and unique animal [62]. Mukherjee et al [23] also emphasized a need to protect and conserve its germplasm in the region through appropriate policy decisions and action. The findings of Qu et al [22] suggest *in*- or *ex-situ* conservation strategies to be effective in halting the further decline of the Mithun population. The *in-* or *ex-situ* conservation was found to have increased the Mithun population from 100 in 1980 to about 3,000 in 2006 in the Yunnan province of China [22]. Thus, countries rearing this animal have an opportunity to embark on *in-situ* and *ex-situ* conservation of germplasm and genetic resources.

This animal suffers from many infectious and non-infec tious diseases [28]. Foot-and-mouth disease (FMD) is the most common viral disease of Mithun with a comparatively higher susceptibility to FMD than other livestock species [64]. The literature on Mithun, regarding their health, is scarce, and detailed information on Mithun diseases and management is not available [64]. Scientific studies are needed to explore the disease prevalence, tolerance, and susceptibility of this animal to develop better health and disease management strategies to reduce mortality and economic losses.

### Transboundary Mithun network for policy lobby and advocacy

Even in the Mithun rearing countries, Chavan et al [65] observed a small number of animals on research farms with limited resources, which reflects weak policy support. Despite a long history of Mithun husbandry, local institutions have not undergone a major change to accommodate new scientific ideas and address modern challenges. Because of the low priority given to institutional development, human resources development has received less attention, which partly explains the slow pace of Mithun research and development. The Mithun rearing countries have yet to come together to share common issues and work out ways to pool scarce resources for achieving common goals. This is probably a primary reason for this unique animal not being able to draw international attention. Knowledge and technologies have not been utilized optimally, largely because Mithun experts and institutions are less aware of each other’s existence in the region. In countries like Bhutan, Mithun research and development had never been led by qualified professionals, thus, room for scientific innovations remains less explored. Nonetheless, the current institutions are making modest efforts to plan and implement programs within their capacity and contribute in a small way to Mithun conservation and development in the region.

Mithun farming is yet to have an enabling environment [62] to encourage investment in entrepreneurship to improve productivity and make this farming sustainable and financially viable. As expressed by Moyong [30], the desirable beef and milk traits of Mithun provide opportunities for preparing different products from the rich composition of its milk. Adding value and diversifying dairy products could enhance the income of ethnic communities. Since Mithun feeds mainly on forest herbs and grass, there is a good opportunity to develop organic products. Further, dairy production could be economic as it is an efficient converter of forest biomass into valued beef, fat milk, and superb leather [30,48], with almost no human inputs besides the provision of an occasional salt lick.

Mithun festivals are likely to be important entry points for future interventions. Opportunities exist to promote cross-border festivals and encourage exchange visits among ethnic groups. In the long run, this shall not only help in preserving the rich Himalayan culture but would also support conservation efforts in the region. It is mainly through festivals that Mithun can be brought to the international limelight and draw global attention. Festivals have the potential to attract international tourists and boost the rural economy in the Eastern Himalaya.

### Development of Mithun institutions in the Eastern Himalaya

Globally, the institutions dedicated to Mithun research and development are scarce, reflecting yet again the weak government policy supports at national, regional, and international levels. Goswami [66] reported that the National Research Centre on Mithun (NRCM) under the Indian Council of Agriculture Research is the only institute in the world dedicated purely to research on conservation and improvement of Mithun. The Centre has two farms namely Medzipheman and Porba farm with a total herd size of 104 and 51 head, respectively [56]. In Bhutan, there are two regional Mithun breeding farms at Wangdigang and Aerong, established in the 1970s under the Department of Livestock. The farms are mandated to breed and distribute pure line Mithun breeding bulls to interested individuals and communities for crossbreeding with local Siri cattle. Currently, there are about 200 pure Mithun in these two farms [34].

In Bangladesh, the Artificial Reproduction Centre for Gayal was established in 1990 at Naikhangchhari, Bandarban district with two breeding bulls and few female gayals [3]. The Mithun Conservation Centre, Lushui, Yunnan province, China was established in 2000 to undertake advanced research (genetic, biochemical, and molecular biology) to generate scientific information for the adoption to further Mithun development and conservation in the field [32]. The institutional network is gradually integrating ethnic communities. Besides, joint research can also be carried out to understand the ecological functions of Mithun, for instance, in shaping the local ecosystem and biodiversity and its roles in maintaining the food chain and population of these large herbivores.

## CONCLUSIONS AND WAY FORWARD

The Mithun is unique species of cattle having socio-cultural and economic significance in the ethnic world. The ecological importance of this bovine species is recognized and has contributed to forest conservation. However, the current population is small and confined to ethnic communities, reared following traditional practices. There are numerous areas for scientific interventions and innovation to enhance Mithun’s productivity and livelihood of ethnic communities. The development of this sacred animal is impeded by weak policies and the legal framework of the respective governments. The relevant institutions are rather weak and institutional linkages are lacking at the regional and international levels to share knowledge, expertise, and exchange information and technologies. There is an urgent need to take stock of the existing situation across Mithun-rearing countries, including infrastructures and human resources, and prepare a concrete roadmap for the conservation and development of this semi-domesticated hill animal. Strong institutions are needed to perform the core functions of Mithun research and development. It is, therefore, critical to building the technical capacity of stakeholders who are expected to build institutional linkages and set up a network among Mithun-rearing countries. Unless concerted efforts are made by the respective governments, the population will decline to a point where no efforts can save this uncommon animal from extinction.

## Figures and Tables

**Table 1:** Distribution (no) and yearly trend of Mithun population

Location	1997^1)^	2003^1)^	2007	2012	2014	2019	2020
India^2)^	176,893	246,315	257,478	297,289	-	386,293	-
Bhutan^3)^	-	-	1,643	570	-	418	-
Myanmar^4)^	-	-	30,000	-	-	-	70,000
Bangladesh	-	-	-	-	-	-	-
China	-	-	-	-	3,000	-	-

1) Ponraj [62].

2) Givernment of India [31].

3) Livestock Statistics [34,50].

4) Zeya [33].

**Table 2:** Mithun rearing tribes in the Eastern Himalayan countries

Tribes rearing Mithun	Districts	States/province	Country
Adi, Adi Gallong, Adi Miyong, Adi Padam, Aka, Apatani, Bangni, Galong, Idu/chulikata Mishmi, Miji, Mishing/Miri, Mishmi, Nissi, Tagin	Anjaw, Dibang valley, Kurung Kumey, Tirap, Lohit, Lower Dibang Valley, Upper Siang, East Siang, West Siang, Upper Subansiri, Lower Subansiri, Papum Pare, East Kameng, West Kameng, Tawang	Arunachal Pradesh	India
Tangkhul, Paite, Any Mizo (Lushai) tribes	Chandel, Ukhrul, Churachandpur, Tamenglong, Senapati	Manipur	India
Chakhesang	Mon, Tuensang, Zunheboto, Dimapur, Kohima, Phek, Longleng, Kiphire, Peren	Nagaland	India
Mizo (Lushai) tribes	Kolasib, Aizawl, Champhai, Serchhip, Saiha	Mizoram	India
Zo ethnic group	Sagaing, Kachin, Chin		Burma
Mru, Bawn, Marmas, Tanchonga	Ruma Upazila, Royangchhari Upazila, Thanchi Upazila (Chittagong and sylhet)	Chittagong hill tracts	Bangladesh
Dulong, Drung	Nujiang	Yunan Province	China
Most farmers from East and East-Central districts	Zhemgang, Samdrup Jongkhar, Chukha, Mongar, Lhuentse, Tashigang, Samtse, Tashiyangtse and Wangdi Phodrang (>10 heads)	East and East-central districts	Bhutan

**Table 3:** Significance and uses of Mithun in ethnic festivals and rituals

Ethnic festival/ritual	Significance of Mithun in the festival	Name of tribe	Place
Ethnic festival			
Solung	Mithun as a symbol of peace and communal harmony	Adi	Arunachal
Mopin	Mithun is sacrificed for good fortune, a successful harvest, and a prosperous new year.	Adi	Arunachal
Reh	Mithun is sacrificed for maintaining the bond of brotherhood and social feelings strong.	Idu Mishmis	Arunachal
Si-Donyi	Mithun is sacrificed to propitiate the Goddess Earth as well as Sun God (Tour My India, 2020).	Tagin	Arunachal
Nyokum Yullo	Mithun is sacrificed to usher in the prosperity and well-being of all living beings.	Nyishi	Arunachal
Etor	Mithun is an expression of gratitude to God for domestic animals.	Adi	Arunachal
Kaquewa	Mithun ox is sacrificed to honor the mountain god.	Dulong	Yunnan
Oros	Mithun is sacrificed during the Muslim religious festival.	Muslim	Bangladesh
Ethnic ritual			
Dotgang	Mithun is sacrificed to bring peace to the soul of the dead.	Adi	Arunachal
Eso Pipak	Mithun to appease God	Adi	Arunachal
Unnying Giidi/sobo	Mithun is sacrificed to prove the masculinity of a man and to bring name and status in society.	Adi	Arunachal
Eso Dorung	Mithun is sacrificed to avert epidemics.	Adi	Arunachal
